# A versatile method for the identification of senolytic compounds

**DOI:** 10.15698/cst2023.12.292

**Published:** 2023-12-11

**Authors:** Chiara Annunziata, Francesca Castoldi, Schlegel Jan, Hazel X. Ang, Mina Ristovska, Stefania Melini, Robert Welch, Christian G. Riedel, Federico Pietrocola

**Affiliations:** 1 Department of Biosciences and Nutrition, Karolinska Institutet, Huddinge, Sweden.; 2 Science for Life Laboratory, Department of Women's and Children's Health, Karolinska Institutet, Solna, Sweden.; 3 Department of Pharmacy, University of Naples Federico II, Naples, Italy.; 4 BioImage Informatics Facility, Science for Life Laboratory, SciLifeLab, Solna, Sweden.

**Keywords:** cell death, senescence, nucleus, fluorescence microscopy, navitoclax

## Abstract

The increased burden of senescent cells is as a well-established hallmark of aging and age-related diseases. This finding sparked significant interest in the identification of molecules capable of selectively eliminating senescent cells, so-called senolytics. Here, we fine-tuned a method for the identification of senolytics that is compatible with high-content fluorescence microscopy. We used spectral detector imaging to measure the emission spectrum of unlabeled control or senescent cells. We observed that senescent cells exhibited higher levels of autofluorescence than their non-senescent counterparts, particularly in the cytoplasmic region. Building on this result, we devised a senolytic assay based on co-culturing quiescent and senescent cells, fluorescently tagged in the nuclear region through the overexpression of H2B-GFP and H2B-RFP, respectively. We validated this approach by showing that first generation senolytics were effective in reducing the number of RFP+ nuclei leaving the count of GFP+ nuclei unaffected. The result was confirmed by flow cytometry analysis of nuclei isolated from these quiescent-senescent cell co-cultures. We found that this system enables to capture cell type-specific effects of senolytics as in the case of fisetin, which kills senescent Mouse Embryonic Fibroblasts but not senescent human melanoma SK-MEL-103 cells. This approach is amenable to genetic and chemical screening for the discovery of senolytic compounds in that it overcomes the limitations of current methods, which rely upon costly chemical reagents or fluorescence microscopy using cells labeled with fluorescent cytoplasmic probes that overlap with the autofluorescence signal emitted by senescent cells.

## INTRODUCTION

The term ‘senescence’ is used to describe a range of cellular states induced in response to sublethal stressors including – but not limited to – DNA damage, CDK4/6 inhibition, oncogene activation and developmental cues [1]. Despite a strong heterogeneity, senescent cells can be identified based on a series of attributes common to different inducers. These encompass (i) a terminal cell cycle arrest (in the case of cells that retain the ability to enter mitosis) accompanied by the overexpression of cyclin-dependent kinase inhibitors such as p16INK4a and p21CIP/WAF1; (ii) the expansion of the lysosomal compartment, characterized by elevated β-galactosidase activity at pH 6.0 (Senescence-Associated β-Galactosidase, SABG); (iii) a secretory phenotype (known as Senescence-Associated Secretory Phenotype, SASP), which reinforces the cell cycle arrest in an autocrine manner and facilitates the paracrine spreading of the senescent phenotype to otherwise non-senescent cells; (iv) the overexpression of anti-apoptotic proteins [1]. The entry into the senescent state involves profound changes in cellular morphology, detectable both at the cellular and subcellular level. It is worth noting that senescent cells undergo a prominent increase in cellular size (reflecting both the enhanced biosynthesis and reduced disposal of metabolic substrates), directly mirrored by an enlargement in the nuclear compartment [2]. In keeping with this notion, a deep learning approach based on the analysis of nuclear morphology has recently been proven sufficient to identify senescent cells *in vitro* and in clinical specimens [3].

A growing body of literature argues for a central role of senescent cells in driving aging and aging-related morbidities, such as metabolic syndromes, frailty, impaired regeneration, fibrosis, and cancer [4]. Indeed, genetic ablation of p16^+^ or p21^+^ cells in mice has shown widespread therapeutic benefits in multiple pathological settings and has been sufficient to extend lifespan and healthspan during natural or accelerated aging [5, 6].

Paralleling the results obtained in genetic mouse models, a number of ‘senolytic’ agents - defined as molecules that induce programmed death of senescent cells without affecting the viability of non-senescent cells - have been identified *in vitro* and validated *in vivo* in a variety of preclinical models [7]. Mechanistically, the ability of senolytics to induce the selective demise of senescent cells has been linked, for example, to the direct inhibition of anti-apoptotic factors that accumulate in senescent cells – as in the case of the Bcl-XL inhibitor navitoclax [8] - or targeting of metabolic pathways required for maintaining senescent cell viability [9]. However, the modes of action for most senolytic agents remain elusive. The increasing interest in expanding the arsenal of senolytics for potential clinical use, along with the unmet need to enhance their specificity and reduce the toxicity, calls for implementation of new strategies to facilitate the discovery of senolytics from compound libraries or genetic screenings. Ideally, such new approach would also expedite shortlisting of senolytics by clarifying whether candidate compounds or genes act in a cell-type or stimuli-specific manner, or rather elicit pan-senolytic effects.

Here, we introduce a fluorescence-based senolytic assay compatible with high-content microscopy or flow cytometry. This method surpasses the constraints of current techniques used for senolytic discovery, eliminating the need for costly chemical reagents to evaluate cellular viability while improving the accuracy of co-culture systems based on the differential fluorescent labelling of quiescent or senescent cells.

## RESULTS AND DISCUSSION

**Figure 1:**
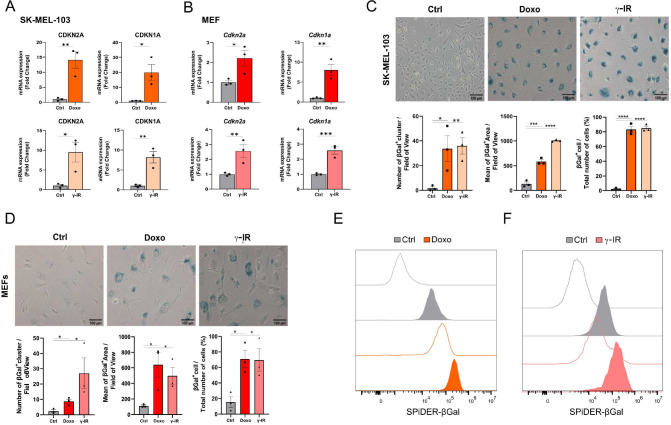
FIGURE 1: Cellular models of senescence. **(A, B)** mRNA expression levels of *CDKN2A* and *CDKN1A* in SK-MEL-103 **(A)** and MEF **(B)** cells untreated or treated with doxorubicin (Doxo) or irradiation (γ-IR). Data are represented as fold change to control (mean ± SEM; pooled from N=3 independent experiments; *p<0.05; **p<0.01; ***p<0.001, unpaired, two-tailed Student's t-test compared to Control). **(C, D)** Representative images and quantification of senescence-associated β-Gal staining of Control, Doxo-treated or γ-IR treated senescent SK-MEL-103 **(C)** and MEF **(D)** cells. Positivity to senescence-associated β-galactosidase is measured as number of ß-Gal positive cluster per field of view, mean of the positive area or percentage of β-Gal^+^ cells over total number of cells. Data are represented as mean ± SEM (pooled from N=3 independent experiments; *p<0.05; **p<0.01; ***p<0.001, ****p<0.0001 unpaired, two-tailed Student's t-test compared to Control). Scale Bar 100 μm. **(E, F)** Flow cytometry analysis of SPiDER-β-Gal fluorescence intensity detected in control or doxo and γ-IR treated senescent SK-MEL-103 **(E)** and MEF **(F)** cells. Transparent histograms indicate the unstained condition per each sample (one representative experiment is shown, N=2 independent experiments).

Cellular senescence is a non-apoptotic response evoked by sub-lethal stress, DNA damage being a prototypical example thereof. In view of the composite effects elicited by exposure to senescence-inducing triggers, the identification of putative senolytic drugs demands the adoption of cellular models where a large fraction of the cell population acquires senescence features. To this end, we used two models of DNA damaged-induced senescence, where senescence was induced by treating human melanoma cells SK-MEL-103 and SV40 large T antigen-immortalized Mouse Embryonic Fibroblasts (MEFs) with the chemotherapeutic doxorubicin (doxo) or γ-irradiation (γ-IR). Successful induction of senescence was confirmed by elevated mRNA expression of the Cyclin-dependent kinase inhibitors CDKN2A and CDKN1A (**Figure 1A, B**) in doxo- and γ-IR-treated cells compared to their untreated controls.

**Figure 2:**
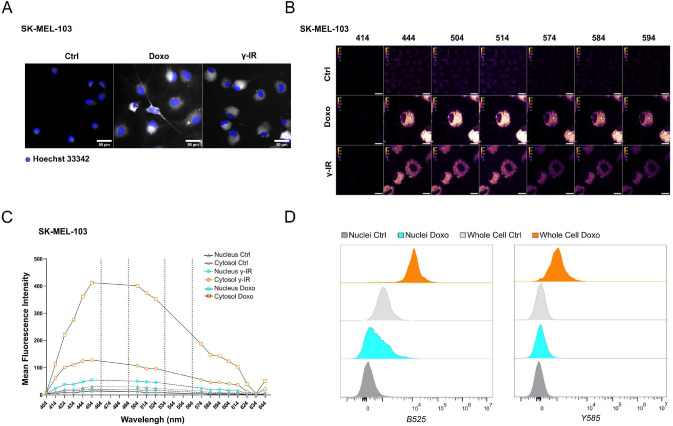
FIGURE 2: Spectral imaging-based profiling of senescent SK-MEL-103 cells. **(A)** Representative images of unlabeled SK-MEL-103 untreated or treated with doxo or γ-IR to induce senescence, acquired with a 488-excitation laser and an emission filter 525/50. Hoechst 33342 was used for nuclear counterstaining. Scale Bar: 50 μm. **(B)** Spectral Imaging analysis of senescent SK-MEL-103 cells, untreated or treated with doxo or γ-IR to induce senescence, after excitation at 405 nm. Representative images recorded at 414 nm, 444 nm, 504 nm, 514 nm, 574 nm, 584 nm, and 594 nm wavelengths are depicted. **(C)** Mean Fluorescence Intensity of the cytoplasmic and nuclear regions of interest of control and senescent SK-MEL-103 cells acquired at wavelengths comprised between 404 nm and 644 nm after excitation at 405 nm. **(D)** Comparative analysis of fluorescence intensity detected in whole control or senescent (doxo-treated) cells or isolated nuclei by flow cytometry. Fluorescence recorded after excitation with blue B525 (left) or yellow Y585 (right) lasers is shown.

Likewise, doxo- and γ-IR-treated cells displayed enhanced SABG activity, as monitored through X-Gal enzymatic assay (**Figure 1C, D**) or detection of Spider-β-Gal fluorescence by flow cytometry (**Figure 1E, F**). Different methodologies were previously employed to identify senolytic targets in low or high throughput setups, including genetic or drug screenings [10]. These assays either rely upon the use of chemical reagents to monitor cellular viability/death or harness fluorescence microscopy [11]. In the latter scenario, non-senescent and senescent cells are stably labeled with two distinct fluorophores and co-cultured. After incubation with candidate compounds, cell counting is employed to identify molecules that selectively induce cell death of senescent cells without significantly affecting the number of non-senescent cells. The induction of senescence entails decreased capacity to recycle and digest lysosomal content through the autophagy pathway, resulting into lipofuscin accumulation [12, 13]. Consistently, unlabeled doxo- and γ-IR-treated senescent cells exhibit increased levels of fluorescence [14] compared to their proliferating counterparts, as confirmed by epifluorescence microscopy (**Figure 2A**; **Supplementary Figure 1A**). Based on this observation, we hypothesized that the intrinsic fluorescence of senescent cells might overlap with the signal emitted from control cells labeled with cytoplasmic fluorescent probes, potentially masking the effect of senolytic drugs that only eliminate a fraction of senescent cells in co-culture settings. To address this limitation, we characterized the autofluorescence spectrum of doxo- and γ-IR-treated unlabeled SK-MEL-103 and MEFs one week after the induction of senescence using spectral imaging. Cells were excited with a 405 nm laser, and fluorescence was detected in 32 channels (**Figure 2B; Supplementary Figure 1B**). Quantitative analysis of the fluorescence spectrum in control and senescent cells was performed in two distinct regions of interest, representing the nuclear and cytosolic compartments (**Figure 2C; Supplementary Figure 1C**). As expected, senescent cells exhibited higher cytoplasmic fluorescence levels, peaking at wavelengths between 454 and 574 nm, in comparison to proliferating cells. However, minimal differences were observed in the nuclear region between senescent and non-senescent cells, which was confirmed by flow cytometry analysis of whole cells and isolated nuclei from both experimental groups (**Figure 2D**). Notably, a shift in fluorescence after excitation with 405 laser was still detectable in nuclei isolated from doxo-treated cells, possibly due to the formation of Senescence-Associated Heterochromatin Foci following DNA damage induction in SK-MEL-103 cells [15]. Intriguingly, even after removing the drug 24 hours after senescence induction, doxo-treated senescent cells exhibited higher levels of fluorescence than irradiated senescent cells. This suggests that the inherent fluorescence of drugs, along with their accumulation in the lysosomes of senescent cells (as previously described for palbociclib [16]), might further intensify fluorescence levels in senescent cells, potentially amplifying or masking signals derived from probes (e.g., antibodies) used to study protein expression in whole senescent cells.

**Figure 3:**
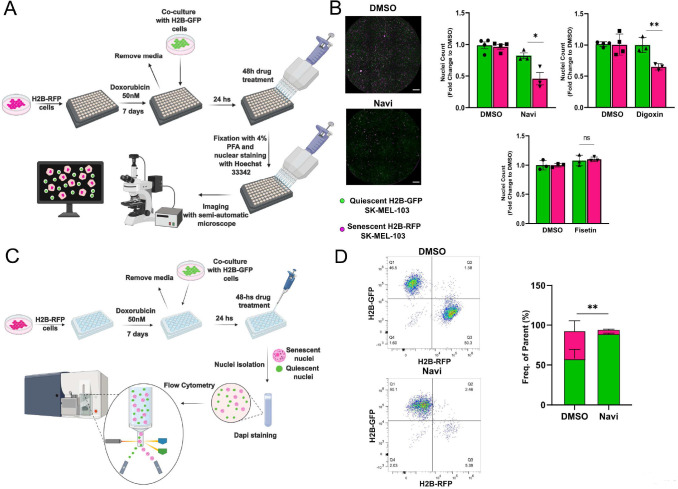
FIGURE 3: Validation of the senolytic assay in SK-MEL-103 cells. **(A)** Workflow of the fluorescence microscopy-based method used to detect senolytic effect of drugs. **(B)** Representative images (left panel) and count (right panel) of H2B-GFP versus H2B-RFP nuclei from H2B-GFP quiescent and H2B-RFP senescent (doxo-treated) SK-MEL-103 cells co-cultures incubated with vehicle (DMSO), navitoclax (Navi), digoxin or fisetin for 48 hours. Data (mean ±SD) are represented as Fold Change to DMSO. One representative experiment (N=3 independent experiments) is shown. (*p< 0.05; **p<0.01, two-tailed Student's t-test compared to senescent DMSO). Scale Bar 500 nm. (C) Workflow of the flow cytometryadapted method used to detect senolytic effect of drugs. (D) Representative density plot (left panel) and quantification (right panel) of H2BGFP and H2B-RFP nuclei isolated from H2B-GFP quiescent/H2B-RFP senescent (doxo-treated) SK-MEL-103 co-cultures, incubated with vehicle (DMSO) or Navi for 48 hours. Data are represented as mean ± SEM (pooled from N=3 independent experiments; **p<0.01, two-tailed Student's t-test compared to senescent DMSO).

To improve the accuracy of senolytics discovery assays, we generated SK-MEL-103 and MEFs cells stably expressing histone H2B tagged with Green Fluorescent Protein (GFP) or Red Fluorescent Protein (RFP). Building on data obtained from spectral imaging, senescence was induced in H2B-RFP transgenic cells by treatment with doxo, while H2B-GFP were used as non-senescent controls. One week after the induction of senescence, H2B-GFP cells were co-seeded at 1:1 ratio with senescent cells, and both cell types were maintained in a 0.01% FBS containing medium to minimize the proliferation of non-senescent cells (**Figure 3A**). Co-cultured cells were then incubated for 48 hours in the presence of vehicle or first-generation senolytics, including navitoclax, digoxin [11], or fisetin [17] (**Figure 3B**). Counting of the differently labeled nuclei was used as a readout to identify any differential killing effects. Consistent with available literature, we found that the pan-senolytic navitoclax significantly reduced the number of senescent – but not quiescent – nuclei in SK-MEL-103 and MEFs (**Figure 3B**; **Supplementary Figure 1D**). Furthermore - aligned with the notion that human cells are more sensitive to cardiac glycosides than murine cells [11] - we confirmed the senolytic effect of digoxin in doxo-treated SK-MEL-103 but not in senescent doxo-treated MEFs. Finally, we observed a cell-type specific effect of fisetin, which induced preferential apoptosis in senescent MEFs yet failed to do so in senescent SK-MEL-103 cells (**Figure 3B**, **Supplementary Figure 1D**). To corroborate the validity of our senolytic assays, we performed flow cytometry of nuclei isolated from the H2B-RFP senescent/H2B-GFP quiescent co-culture (**Figure 3C**), revealing an unaffected frequency of GFP^+^ quiescent nuclei and a reduced frequency of RFP^+^ senescent nuclei upon treatment with navitoclax (**Figure 3D**).

Overall, the results presented here highlight approaches based on differential nuclear staining of non-senescent or senescent cells as a rapid and cost-effective strategy for identifying senolytic agents. This method is ideally suited for screening chemical libraries using high-content fluorescence microscopy or flow cytometry. We propose that this method increases the precision of co-culture-based senolysis assays, enabling the detection of low-magnitude effects elicited by senolytic agents that only eliminate a fraction of senescent cells.

## MATERIALS AND METHODS

### Cell Culture

SK-MEL-103 (human melanoma) and HEK293T (Human Embryonic Kidney) cells were obtained from ATCC. The SV40 large T antigen-immortalized mouse embryonic fibroblast (MEF) cell line was generated by Dr. Mervi T. Hyvönen, School of Pharmacy, University of Eastern Finland (Kuopio). All cell lines were cultured in standard DMEM (Gibco), supplemented with 10% heat inactivated FBS (Gibco) and 1% antibiotics (penicillin/streptomycin 100 U/mL, Gibco). Cells were maintained in a humidified incubator at 37°C and 5% CO_2_. Cells were bi-weekly tested for mycoplasma contamination using Mycoalert™ Mycoplasma Detection Kit (Lonza), and only negative cells were used for experiments.

Senescence was induced by treatment with the DNA-damage inducing agent doxorubicin (50 nM in SK-MEL-103; 150 nM in MEFs, Sigma Aldrich) or by γ-IR (7 Gy in SK-MEL-103; 10 Gy in MEFs). An equal number of untreated control cells were seeded at day 6 after senescence induction and harvested along with senescent cells on day 7 after treatment for further analysis.

For senolysis induction, control or senescent cells were treated for 48 hours with DMSO (vehicle), navitoclax (1 µM, Tebu-Bio #T2101), digoxin (100 nM, Sigma-Aldrich, #D6003), fisetin (10 µM, Tocris, # 5016/50). For the co-culture assay, H2B-RFP cells (6000-8000 cells/well) were seeded in 96-well glass-bottom plates for imaging (MoBiTec GmbH, #524220). Doxo was added 24 hours after seeding to induce senescence. 6 days after the induction of senescence, control H2B-GFP cells (6000–8000 cells/well) were co-seeded in DMEM supplemented with 0.01% of FBS to keep them in a quiescent state. At day 7 after senescence induction, cells were incubated with senolytic drugs for 48 hours. After treatment, cells were fixed with 4% v/v PFA solution containing Hoechst 33342 (Invitrogen, #H3570) for 20′ at room temperature and washed twice with PBS prior to image acquisition.

### Real Time-qPCR

RNA was extracted from cellular lysates using Trizol (Invitrogen, #12034977) and quantified using a Qubit fluorometer. cDNA was produced from total RNA using the iScript cDNA Synthesis Kit (Bio-Rad, #1708889) according to manufacturer instructions. Real-time PCR was performed with GoTaq PCR Master Mix (Promega, #A6002) for the following genes using the stated primers:

### Senescence Associated Beta Galactosidase Assay

Staining was performed as previously described in [18]. Briefly, cells were washed with Phosphate Buffered Saline (PBS) and fixed in 0.2% of glutaraldehyde solution. Cells were washed with PBS and stained with a β-Galactosidase (β-Gal) solution prepared by dissolving 1 mg/mL 5-Bromo-4-Chloro-3-Indolyl β-D-Galactopyranoside (X-Gal; #203782, Sigma-Aldrich) in dimethylformamide at pH 6. Cells were kept overnight at 37˚C and then washed with PBS before imaging. Images were acquired using a brightfield microscope.

### Transgenic cell line generation

To produce lentivirus, HEK293T cells (2.5×10^6^) were seeded in a p100 dish and were transfected with 5 µg lentiviral pHIV-H2BmRFP (Addgene, ID:18982) or pHIV-H2B-eGFP (Addgene, ID: 91776), 6 µg psPAX2 (Addgene, ID: 12260) and 2 µg pMD2.G (Addgene, ID 12259) plasmids using the FuGENE HD transfection reagent (#E2312, Promega). The supernatant was filtered through a 0.45 µm membrane filter and used to infect recipient SK-MEL-103 or MEFs cells (1x10^6^) in a p100. Infection media was changed twice *per* day (8/10 hours of incubation for each infection), for two days. Polybrene (8 µg/mL, Sigma Aldrich #H9268) was used to enhance transduction efficacy.

### Flow Cytometry

Cells were harvested, centrifuged at 1200 rpm for 5′ and washed twice with PBS. Assessment of SABG activity by flow cytometry was performed using the SPiDER-β-Gal probe (Dojindo Molecular Technologies, Inc. #SG02-10), according to manufacturer instruction. Briefly, cells were harvested and pre-treated with Bafilomycin A1 (Med Chem Express, 100 nM) for 30' before incubation with 10 mM SPiDER-ß-Gal for 1 hour. Cells were incubated with the viability dye DAPI (#D9542, Sigma) at 5 µg/mL concentration for 30′ at room temperature (RT) and analyzed with a Cytoflex (Beckman Coulter) flow cytometer. For the senolytic assay, co-culture experiment was executed as described above. At the end of the experiment, nuclei were isolated from H2B-GFP/H2B-RFP co-cultures using the Nuclei Isolation Kit (Sigma Aldrich, #NUC101) as per manufacturer instructions. Nuclei were resuspended in PBS containing 5mM EDTA and 0.5% BSA and stained with DAPI at 5µg/mL final concentration prior to flow cytometry analysis. Data were analyzed using FlowJo software v.10.9.0.

### Microscopic images

For the co-culture experiment, images were acquired in widefield acquisition using a Nikon Ti2 inverted microscope equipped with Kinetix sCMOS Camera (>95% QE, 6.5 µm pixel sizes, 2720x2720 pixels). The JOBS module for high content screening (Nikon NIS Element software) was used to run the image acquisition process with an automatic stage. A large image (3x3) was acquired to capture the whole well. Each tile was automatically stitched via blending in NIS element software. Spectral imaging and the acquisition of unlabeled SK-MEL-103 and MEF were performed using a single-point scanning confocal microscope (Nikon) equipped with a 32/channel spectral detector using 405, 488, and 561 lasers for excitation along with the same dichroic mirrors (405/488/561/640) per each image. The images acquired with the 405 lasers were used for the analysis. The spectrum for each region of interest (ROI) was obtained by plotting the intensity over the spectral stack using ImageJ. Analysis and quantification of the co-culture plates was run using a Python script available online at https://github.com/BIIFSweden/SenolyticDetection. To quantify the SABG activity from the acquired brightfield image we used a custom-written ImageJ macro available online at https://github.com/the-biking-viking/SAbG-Assay. In brief, the macro converts the brightfield images into a 3-slice stack consisting of hue, saturation, and brightness (HSB). The active blue SABG clusters are detected by thresholding and masking each of the individual images in the HSB stack and combining the overlapping parts of the masks. On this final overall mask, each cluster is detected by using the “Analyze Particles” command of ImageJ, and the “intensity” of each detected cluster is measured in the converted saturation image of the raw image. The list with each detected cluster, the detected regions of interest, the total mask and the converted saturation image are finally saved in a results folder of the selected raw data.

### Statistical analysis

Data were analyzed using GraphPad Prism v9.3.0. Statistical analyses were performed as indicated in the corresponding figures. Differences were considered significant based on *p*-values (*p<0.05; ** *p* <0.01; *** *p* <0.001, **** *p* <0.0001).

## AUTHOR CONTRIBUTION

C. Annunziata: conceptualization, data curation, formal analysis, validation, investigation, methodology, supervision, visualization, writing-original draft, reviewing and editing; F. Castoldi: formal analysis, validation, investigation, reviewing and editing; J. Schlegel: data curation, formal analysis, methodology, software, visualization, reviewing and editing; H. X. Ang: conceptualization, validation, investigation, reviewing and editing; M. Ristovska: investigation, validation; S. Melini: investigation, validation; R. Welch: methodology, software; C. G. Riedel: conceptualization, validation, reviewing and editing; F. Pietrocola: conceptualization, resources, data curation, formal analysis, supervision, funding acquisition, writing-original draft, project administration, writing-review and editing. All the authors discussed the results and commented on the manuscript.

## SUPPLEMENTAL MATERIAL

Click here for supplemental data file.

All supplemental data for this article are available online at www.cell-stress.com/researcharticles/2023a-annunziata-cell-stress/.

## Abbreviations:

γ-IR: – γ-irradiation,
doxo: – doxorubicin,
GFP: – green fluorescent protein,
MEF: – mouse embryonic fibroblasts,
RFP: – red fluorescent protein,
SABG: – senescence-associated β-galactosidase.

## References

[1] Hernandez-Segura A, Nehme J, Demaria M (2018). Hallmarks of Cellular Senescence.. Trends Cell Biol.

[2] Kusumoto D, Seki T, Sawada H, Kunitomi A, Katsuki T, Kimura M, Ito S, Komuro J, Hashimoto H, Fukuda K, Yuasa S (2021). Anti-senescent drug screening by deep learning-based morphology senescence scoring.. Nat Commun.

[3] Heckenbach I, Mkrtchyan GV, Ezra MB, Bakula D, Madsen JS, Nielsen MH, Oro D, Osborne B, Covarrubias AJ, Idda ML, Gorospe M, Mortensen L, Verdin E, Westendorp R, Scheibye-Knudsen M (2022). Nuclear morphology is a deep learning biomarker of cellular senescence.. Nat Aging.

[4] Munoz-Espin D, Serrano M (2014). Cellular senescence: from physiology to pathology.. Nat Rev Mol Cell Biol.

[5] Baker DJ, Wijshake T, Tchkonia T, LeBrasseur NK, Childs BG, van de Sluis B, Kirkland JL, van Deursen JM (2011). Clearance of p16Ink4a-positive senescent cells delays ageing-associated disorders.. Nature.

[6] Wang B, Wang L, Gasek NS, Zhou Y, Kim T, Guo C, Jellison ER, Haynes L, Yadav S, Tchkonia T, Kuchel GA, Kirkland JL, Xu M (2021). An inducible p21-Cre mouse model to monitor and manipulate p21-highly-expressing senescent cells in vivo.. Nat Aging.

[7] Niedernhofer LJ, Robbins PD (2018). Senotherapeutics for healthy ageing.. Nat Rev Drug Discov.

[8] Chang J, Wang Y, Shao L, Laberge RM, Demaria M, Campisi J, Janakiraman K, Sharpless NE, Ding S, Feng W, Luo Y, Wang X, Aykin-Burns N, Krager K, Ponnappan U, Hauer-Jensen M, Meng A, Zhou D (2016). Clearance of senescent cells by ABT263 rejuvenates aged hematopoietic stem cells in mice.. Nat Med.

[9] Johmura Y, Yamanaka T, Omori S, Wang TW, Sugiura Y, Matsumoto M, Suzuki N, Kumamoto S, Yamaguchi K, Hatakeyama S, Takami T, Yamaguchi R, Shimizu E, Ikeda K, Okahashi N, Mikawa R, Suematsu M, Arita M, Sugimoto M, Nakayama KI, Furukawa Y, Imoto S, Nakanishi M (2021). Senolysis by glutaminolysis inhibition ameliorates various age-associated disorders.. Science.

[10] Power H, Valtchev P, Dehghani F, Schindeler A (2023). Strategies for senolytic drug discovery.. Aging Cell.

[11] Triana-Martinez F, Picallos-Rabina P, Da Silva-Alvarez S, Pietrocola F, Llanos S, Rodilla V, Soprano E, Pedrosa P, Ferreiros A, Barradas M, Hernandez-Gonzalez F, Lalinde M, Prats N, Bernado C, Gonzalez P, Gomez M, Ikonomopoulou MP, Fernandez-Marcos PJ, Garcia-Caballero T, Del Pino P, Arribas J, Vidal A, Gonzalez-Barcia M, Serrano M, Loza MI, Dominguez E, Collado M (2019). Identification and characterization of Cardiac Glycosides as senolytic compounds.. Nat Commun.

[12] Georgakopoulou EA, Tsimaratou K, Evangelou K, Fernandez Marcos PJ, Zoumpourlis V, Trougakos IP, Kletsas D, Bartek J, Serrano M, Gorgoulis VG (2013). Specific lipofuscin staining as a novel biomarker to detect replicative and stress-induced senescence. A method applicable in cryo-preserved and archival tissues.. Aging.

[13] Evangelou K, Lougiakis N, Rizou SV, Kotsinas A, Kletsas D, Munoz-Espin D, Kastrinakis NG, Pouli N, Marakos P, Townsend P, Serrano M, Bartek J, Gorgoulis VG (2017). Robust, universal biomarker assay to detect senescent cells in biological specimens.. Aging Cell.

[14] Bertolo A, Baur M, Guerrero J, Potzel T, Stoyanov J (2019). Autofluorescence is a Reliable in vitro Marker of Cellular Senescence in Human Mesenchymal Stromal Cells.. Sci Rep.

[15] Aird KM, Zhang R (2013). Detection of senescence-associated heterochromatin foci (SAHF).. Methods Mol Biol.

[16] Llanos S, Megias D, Blanco-Aparicio C, Hernandez-Encinas E, Rovira M, Pietrocola F, Serrano M (2019). Lysosomal trapping of palbociclib and its functional implications.. Oncogene.

[17] Yousefzadeh MJ, Zhu Y, McGowan SJ, Angelini L, Fuhrmann-Stroissnigg H, Xu M, Ling YY, Melos KI, Pirtskhalava T, Inman CL, McGuckian C, Wade EA, Kato JI, Grassi D, Wentworth M, Burd CE, Arriaga EA, Ladiges WL, Tchkonia T, Kirkland JL, Robbins PD, Niedernhofer LJ (2018). Fisetin is a senotherapeutic that extends health and lifespan.. EBioMedicine.

[18] Dimri GP, Lee X, Basile G, Acosta M, Scott G, Roskelley C, Medrano EE, Linskens M, Rubelj I, Pereira-Smith O (1995). A biomarker that identifies senescent human cells in culture and in aging skin in vivo.. Proc Natl Acad Sci U S A.

