# Supplementary material for: A versatile method for the identification of senolytic compounds
Source: Cell Stress. 2023 Dec 11;7(12):105–11. doi: 10.15698/cst2023.12.292 (PMC10742873; doi:10.15698/cst2023.12.292)
Supplement: Supplementary file 1 [file ces-07-105-s01.pdf]

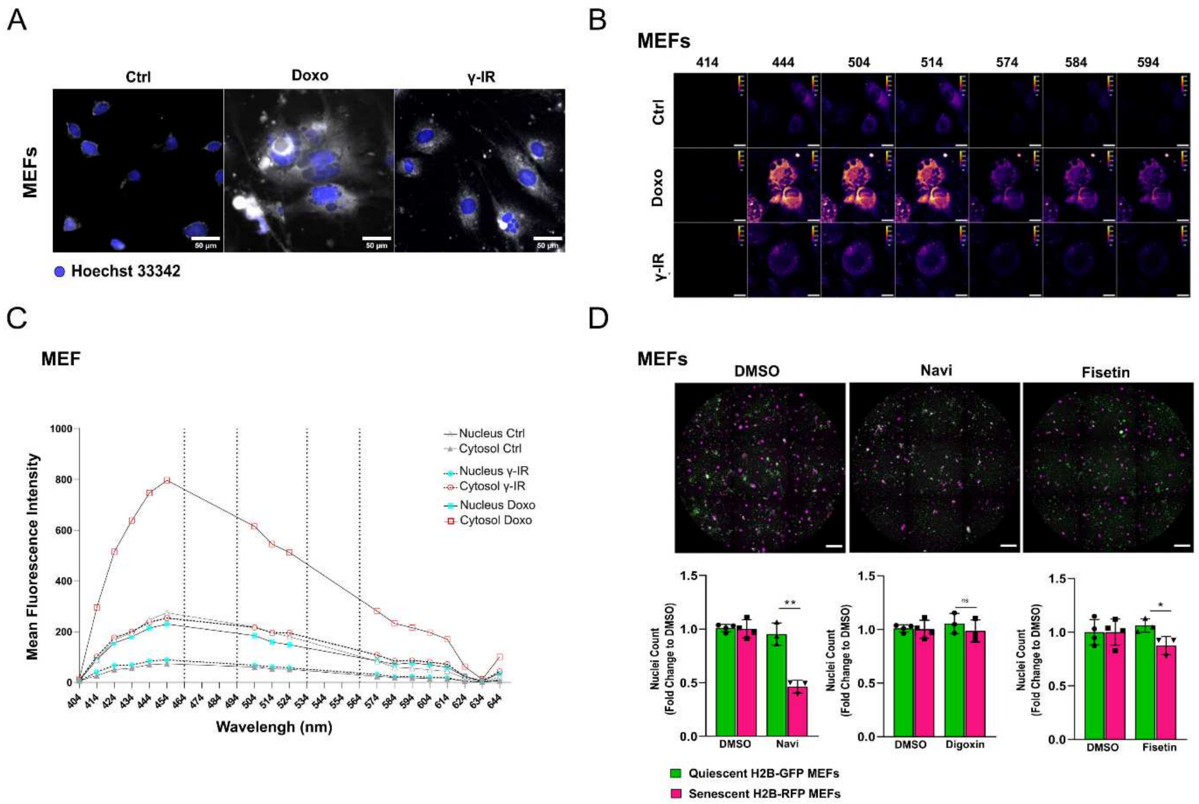

Supplemental FIGURE 1: Spectral imaging-based and senolytic assay in MEFs. (A) Representative images of unlabeled MEFs untreated or treated with doxo or γ-IR to induce senescence, acquired with a 488-excitation laser and an emission filter 525/50. Hoechst 33342 was used for nuclear counterstaining. Scale Bar: 50 µm. **(B)** Spectral Imaging analysis of senescent MEFs, untreated or treated with doxo or γ-IR to induce senescence, after excitation at 405nm. Representative images recorded at 414nm, 444nm, 504nm, 514nm, 574nm, 584nm, and 594nm wavelengths are depicted. **(C)** Mean Fluorescence Intensity of the cytoplasmic and nuclear regions of interest of control and senescent MEFs, acquired at wavelengths comprised between 404nm and 644nm after excitation at 405nm. **(D)** Representative images and count of H2B-GFP versus H2B-RFP nuclei from H2B-GFP quiescent and H2B-RFP senescent (doxo-treated) MEF cells co-cultures incubated with vehicle (DMSO), navitoclax (Navi), digoxin or fisetin for 48 hours. Data (mean ± SD) are represented as Fold Change to DMSO. One representative experiment (N= 3 independent experiments) is shown (**p*< 0.05; ***p*<0.01, two-tailed Student's t-test compared to senescent DMSO). Scale Bar: 500 nm.
